# Deep Learning-Based Monocular Depth Estimation Methods—A State-of-the-Art Review

**DOI:** 10.3390/s20082272

**Published:** 2020-04-16

**Authors:** Faisal Khan, Saqib Salahuddin, Hossein Javidnia

**Affiliations:** 1 College of Engineering and Informatics, National University Ireland Galway, Galway H91 TK33, Ireland; f.khan4@nuigalway.ie (F.K.); saqib.salahuddin@nuigalway.ie (S.S.); 2 ADAPT Centre, Trinity College Dublin, Dublin D02 PN40, Ireland

**Keywords:** monocular depth estimation, single image depth estimation, CNN monocular depth

## Abstract

Monocular depth estimation from Red-Green-Blue (RGB) images is a well-studied ill-posed problem in computer vision which has been investigated intensively over the past decade using Deep Learning (DL) approaches. The recent approaches for monocular depth estimation mostly rely on Convolutional Neural Networks (CNN). Estimating depth from two-dimensional images plays an important role in various applications including scene reconstruction, 3D object-detection, robotics and autonomous driving. This survey provides a comprehensive overview of this research topic including the problem representation and a short description of traditional methods for depth estimation. Relevant datasets and 13 state-of-the-art deep learning-based approaches for monocular depth estimation are reviewed, evaluated and discussed. We conclude this paper with a perspective towards future research work requiring further investigation in monocular depth estimation challenges.

## 1. Introduction

Monocular depth estimation is a fundamental challenge in computer vision and has potential applications in robotics, scene understanding, 3D reconstruction and medical imaging [1,2,3,4]. This problem remains challenging as there are no reliable cues for perceiving depth from a single image. For example, temporal information and stereo correspondences are missing from such images. The classical depth estimation approaches heavily rely on multi-view geometry [5,6,7,8,9] such as stereo image [10,11]. These methods require alignment and calibration procedures which are important for multi-camera or multi-sensor depth measurement systems [12,13]. Multi-view methods acquire depth information by utilising visual cues and different camera parameters.

Most of the binocular or multi-view methods are able to estimate fairly accurate depth information. However, their computational time and memory requirements are important challenges for many applications [14]. The idea of using the monocular image to capture depth information could potentially solve the memory requirement issue, but it is computationally difficult to capture the global properties of a scene such as texture variation or defocus information.

Recently, the advancement of Convolutional Neural Networks (CNN) and publicly available datasets have significantly improved the performance of monocular depth estimation methods [15,16,17,18,19].

This paper offers a comprehensive and structured survey of deep learning-based monocular depth estimation approaches. The goal of the review is to assist the reader to navigate this emerging field, which has become of significant interest to the computer vision community in recent years. The rest of the survey is organized as follows: Section 2 presents a summary and basic concept of monocular depth estimation, problem description, traditional methods for depth estimation and publicly available datasets. Section 3 reviews the recent deep learning architectures for monocular depth estimation categorised in supervised, self-supervised and semi-supervised methods. Section 4 compares the state-of-the-art approaches followed by discussion and potential future research directions presented in Section 5.

## 2. An Overview of Monocular Depth Estimation

The concept of depth estimation refers to the process of preserving 3D information of the scene using 2D information captured by cameras. Monocular solutions tend to achieve this goal using only one image. These methods aim to estimate distances between scene objects and the camera from one viewpoint. This requires the method to perform depth estimation on low-cost embedded systems. There are a variety of devices commercially available to provide depth information, however, their processing power, computational time, range limitation and cost make them impractical for consumer devices. Sensors such as Kinect are commonly used in consumer devices [20,21]. These types of sensor are categorized as Time-of-Flight (ToF) where the depth information is acquired by calculating the time required for a ray of light to travel from a light source to an object and back to the sensor [22]. ToF sensors are more suitable for the indoor environment and short range (<2 m) depth sensing. On the other hand, laser-based scanners (LiDAR) are commonly utilised for 3D measurement in the outdoor environment. The key advantages of LiDAR sensors are high resolution, accuracy, performance in low light and speed. However, LiDARs are expensive devices and they require extensive power resources which make them unsuitable for consumer products.

It has been shown in the state-of-the-art that monocular depth estimation methods could be a potential solution to address many of these challenges [23,24,25]. These methods perform with a relatively small number of operations and in less computation time. They do not require alignment and calibration which is important for multi-camera, or multi-sensor depth measurement systems. Accurate monocular depth estimation methods can play an important role in understanding 3D scene geometry and 3D reconstruction, particularly in cost-sensitive applications and use cases.

### 2.1. Problem Representation

Let $I\in {\mathbb{R}}^{w\times h}$ be an image with size w×h. The goal is to estimate the corresponding depth information $D\in {\mathbb{R}}^{w\times h}$. This is an ill-posed problem as there is an ambiguity in the scale of the depth. Supervised learning-based methods try to address this issue by approximately learning the scale from a set of training images. On the other hand, unsupervised and semi-supervised methods often utilise an extra input for training such as stereo image sets, visual odometry and 6D camera pose estimation to tackle the scale ambiguity issue. These methods mathematically define the problem as follows: given a large dataset of Red-Green-Blue (RGB) and depth images, single image depth estimation can be considered as a regression problem that uses a standard loss function such as Mean Square Error (MSE). To achieve this, a training set τ can be represented as follows:

(1) $$\tau =\left\{\left(I_{n},D_{n}\right)\right\},I_{n}\in {\mathbb{R}}^{w\times h}\;and\;D_{n}\in {\mathbb{R}}^{w\times h}$$

### 2.2. Traditional Methods for Depth Estimation

Most of the traditional methods for depth estimation rely on the assumption of having observations of the scene, either in space or time (e.g., stereo or multi-view, structure from motion) [10,11,26,27]. Traditional methods can be categorized in two sets, active and passive methods.

Active methods involve computing the depth in the scene by interacting with the objects and the environment. There are different types of active method, such as light-based depth estimation, which uses the active light illumination to estimate the distance to different objects. Ultrasound and ToF are other examples of active methods. These methods use the known speed of the wave to measure the time an emitted pulse takes to arrive at an image sensor. Passive methods exploit the optical features of captured images. These methods involve extracting the depth information by computational image processing. In the category of passive methods, there are two primary approaches: (a) multi-view depth estimation, such as depth from stereo, and (b) monocular depth estimation.

The traditional depth estimation methods are mainly focused on multi-view geometry. The detailed review of those methods is outside the scope of this work. However, it is worth noting that multi-view traditional methods have various limitations including computational complexity and associated high energy requirements. Current research works take advantage of deep-learning methods to achieve more accurate results with lower computational and energy demands [15,16,17,18,19]. Deep learning-based approaches and the availability of large-scale datasets have significantly transformed the monocular depth estimation methods.

### 2.3. Datasets for Depth Estimation

A number of important datasets are particularly preferred for the depth estimation problem as they provide images and corresponding depth maps from different viewpoints. The following section highlights the popular datasets used to analyse the scenes. Consumer-level sensors such as the Kinect and Velodyne laser scanner [20,21,28] are commonly used to capture the ground truth depth images for datasets. A summary is presented in Table 1.

**NYU-v2:** the NYU-v2 dataset for depth estimation was introduced in [29]. The dataset consists of 1449 RGB images densely labelled with depth images. The datasets consist of 407K frames of 464 scenes taken from three different cities. These datasets are used for indoor scenes depth estimation, segmentation and classification.
**Make3D:** the Make3D dataset, introduced in [30], contains 400 and 134 outdoor images for training and testing, respectively. This dataset contains different types of outdoor, indoor and synthetic scenes that are used for depth estimation by presenting a more complex set of features.
**KITTI:** the KITTI dataset, introduced in [31], has two versions and is made of 394 road scenes providing RGB stereo sets and corresponding ground truth depth maps. The KITTI dataset is further divided into RD: KITTI Raw Depth [31]; CD: KITTI Continuous Depth [31,32]; SD: KITTI Semi-Dense Depth [31,32]; ES: Eigen Split [33]; ID: KITTI Improved Depth [34]. KITTI datasets are commonly used for different tasks including 3D object detection and depth estimation. The high-quality ground truth images are captured using the Velodyne laser scanner.
**Pandora:** the Pandora dataset, introduced [35], contains 250K full resolution RGB and corresponding depth images having their corresponding annotation. Pandora dataset is used for head centre localization, head pose estimation and shoulder pose estimation.
**SceneFlow:** this was introduced in [36] as one of the very first large-scale synthetic datasets consist of 39K stereo images with corresponding disparity, depth, optical flow and segmentation masks.

## 3. Deep Learning and Monocular Depth Estimation

There has been a significant improvement in learning-based monocular depth estimation methods over the past couple of years [37,38,39,40,41,42]. The majority of the deep learning-based methods involve a CNN trained on RGB-images and the corresponding depth maps. These methods can be categorized into supervised, semi-supervised and self-supervised. Supervised methods accept a single image and the corresponding depth information for training. In such a case, the trained network can directly output the depth information. However, a large amount of high-quality depth data is required, which is hard to generalize to all use cases.

To overcome the need for high-quality depth estimation as seed data, numerous semi-supervised methods are proposed. Semi-supervised approaches require smaller amount of labelled data and a large amount of unlabeled data for training [16,43,44]. The limitation of semi-supervised methods is that the networks are unable to correct their own bias and require additional domain information such as camera focal length and sensor data.

Self-supervised methods only require a small number of unlabeled images to train the networks for depth estimation [15,42,45]. These methods obtain the depth information automatically by relating different input modalities. Self-supervised methods suffer from generalization issues. The models can only perform on a very limited set of scenarios with similar distribution as the training set.

Table 2 categorizes thirteen methods reviewed comprehensively in the next sub-sections into supervised, semi-supervised and self-supervised.

### 3.1. Supervised Methods

Rosa et al. [32] proposed a supervised framework to estimate continuous depth maps from LiDAR points. The framework utilises Hilbert Maps methodology [55] to generate dense depth map from the sparse point could projected from LiDAR scanner. Furthermore, the proposed framework takes advantage of the Fully Convolutional Residual Network (FCRN) proposed by Laina et al. [56] for depth estimation. The network is trained on the densified depth images which are augmented by flipping and applying colour distortion. Despite the comparable performance of this method against the state-of-the-art methods, it can only produce depth maps with 128×160 pixel resolution. More importantly, the network is biased by the output of the Hilbert maps’ densification process which does not represent the truth depth information of the missing areas.

Yuru et al. [46] proposed a new supervised algorithm called the Attention-Based Context Aggregation Network (ACAN) to estimate depth maps. The algorithm utilises the deep residual architecture [57], dilated layer and self-attention module [58,59,60] to control the spatial scale and continuous pixel-level dense depth estimation. Moreover, the self-attention module creates a relationship among every pixel resulting in learning the attention weights and contextual information which can produce more accurate depth information. Furthermore, the algorithm uses image-pooling to combine the image-level information for depth estimation. Soft-ordinal inference translation is used to transform the predicted probabilities into continuous depth values to produce more realistic depth maps. The network is trained on resized and cropped images from NYU-v2 [29] and KITTI [31] datasets. The context adaption feature of this network results in sharp boundaries in the structure of the predicted depth map.

Ibraheem et al. [47] proposed a supervised method to estimate depth maps with the help of transfer learning. The method utilises a CNN for estimating high-quality depth maps. The method uses standard encoder-decoder network architecture based on pre-trained DenseNet-169 [61] and ImageNet [62] networks for features extraction. Furthermore, the information obtained is passed to the decoder to calculate the final depth maps with the sampling layer [63]. The network is trained on the densified depth images, which are augmented by horizontal flipping and applying the colour distortion including swapping the green and red channels of the input images. It produces depth maps with 320×240 pixel resolution and is likely to be biased by the output of the bilinear upsampling layer which does not represent the accurate depth information for all regions.

Fu et al. [18] proposed a supervised method to estimate depth maps from the Spacing-Increasing Discretization (SID) approach. The framework utilises the dense feature extractor, cross channel information learner, multi-scale feature learner, encoder and ordinal regression optimizer for high-quality depth estimation. Furthermore, the network is defined in a simpler way that avoids needless subsampling and captures multi-scale information to save computational cost and time. The subsampling layers are removed in the pooling layers and dilated convolutions are added to obtain more accurate depth information. The network is trained on four challenging datasets including Make3D [30], NYU-v2 [29], KITTI [31] and ScanNet [64] to introduce more feature variations.

Yin et al. [48] proposed a supervised framework to estimate depth maps by taking advantage of the 3D geometric constraints. A simple type of geometric constraints known as ‘virtual norm’ is implemented which is determined by randomly sampled three points in the 3D reconstruction to obtain a high-quality depth estimation. Further, the method can estimate 3D structures of the scene and surface normals directly from depth maps.

The method uses the 3D geometric constraints to convert the estimated depth to 3D point cloud representations. The network is trained on the densified depth images which are augmented by randomly cropping and flipping. This method can produce depth maps with 384×512 pixel resolution which are more robust and have strong global constraints.

Jin et al. [49] proposed a supervised method for monocular depth estimation that uses new Local Planar Guidance Layers (LPGL) inserted into the decoding phase of the network. The method utilises a decoding stage with spatial resolutions of 1/8, 1/4 and 1/2 by placing a layer that guides the input features to the desired depth. Furthermore, a Dense Feature Extractor (DFE), Contextual Information Extractor (CIE), LPGL and their dense features are used for final depth estimation. The proposed framework takes advantage of the dense Atrous Apatial Pyramid Pooling layer [65] for depth estimation. The network is trained on random crop of size 352×704 for KITTI [31] and 416×544 for NYU-v2 [29] datasets.

Zachary et al. [50] targeted the issues of monocular depth estimation in videos. The proposed method known as DeepV2D combines two classical algorithms in an end-to-end architecture. The network consists of two modules, depth estimation and camera motion. The depth module takes the camera motion as input and returns an initial depth map. The camera motion module takes the predicted depth and outputs the refined camera motion. Furthermore, the network alternates between these two modules to predict the final depth map. The network is trained on four challenging datasets including Make3D [30], NYU-v2 [29], KITTI [31] and ScanNet [64] to introduce more feature variations and high quality depth estimation.

### 3.2. Self-Supervised Methods

Matan et al. [51] proposed a self-supervised method to estimate depth maps from Siamese networks [66] approaches. The method utilises the Siamese DispNet [36], ResNet [57] and VGG [67] based network architectures for depth estimation. Further, the method predicts multi-scale disparity maps in four scales which are later concatenated with previous decoder layer output and the corresponding encoder output using the skip connections. The network is trained on the RGB and ground truth depth images with 1242×375 pixel resolution. The proposed network has the advantage of sharing weights to reduce computational operations by cutting the network size to half which could lead to a potential model for consumer devices.

Aleotti et al. [38] proposed a self-supervised framework to estimate depth maps using end-to-end monocular residual matching known as monoResMatch. The framework utilises stereo matching approach for depth estimation. The RGB image is mapped to the feature space and then synthesized to obtain features aligned with virtual right images. The network further considers high dimensional features at input image resolution to find multi-scale inverse depth map aligned with the input image. The model is constructed based on an hourglass structure with skip connections. The final stage consists of a disparity refinement module which estimates residual corrections to the initial disparity. The network is trained using Structural Similarity (SSIM) reconstruction loss, disparity smoothness loss with an edge-aware term and reverse Huber loss [68]. The model is trained on Cityscape [69] and KITTI [31] datasets with random crops of size 640×192.

Guizilini et al. [52] proposed a self-supervised method to estimate depth maps by combining the geometry of the PackNet. The method utilises the symmetrical packing and unpacking blocks to combine the encoded and decoded information using 3D convolutions. The network follows a similar architecture as [70], which provides the encoder-decoder layers with skip connections having geometrical information of the dense depth estimation. Furthermore, the method introduces new packing and unpacking blocks having visual information for fine-grained high-resolution depth predictions. This model is trained on the RGB and ground truth depth images with 640×192 pixel resolution from unlabelled data which can be generalized into unseen environments. The proposed architecture uses upsampling and downsampling operations which increase the number of the parameters and result in inaccurately scaled depth maps.

Andraghetti et al. [53] employed a state-of-the-art visual odometry method to obtain 3D points and sparse depth maps. Furthermore, the sparse data is fed to a sparse auto-encoder to obtain a denser depth map. The output of this stage along with the corresponding RGB image are fed to a CNN to acquire a final densified depth map in a self-supervised manner. The network is trained on the RGB and ground truth depth images from the KITTI [31] dataset and predicts depth maps with 256×512 pixel resolution.

Clement et al. [42] proposed a self-supervised approach to estimate depth maps utilising a combination of three architectures and loss functions. The pipeline takes advantage of a fully connected U-Net [71] to predict depth and a pose network to estimate the pose between pairs of images. ResNet-18 [57] is selected as the encoder and the pre-trained ImageNet [62] model is used to initialise the weights. The proposed framework utilises appearance-based loss and it introduces a modified per-pixel minimum reprojection loss. The network is trained on KITTI [31] dataset with Eigen split and it estimate depth maps with 640×192 pixel resolution.

### 3.3. Semi-Supervised Methods

Shanshan et al. [54] proposed GASDA, a semi-supervised method to estimate depth maps using the geometry-aware symmetric domain adaption. This approach targets the generalisation issue of the depth estimation methods by training the model on synthetic data to estimate depth from natural images. The method uses symmetric style image translation and monocular depth prediction. Utilising the CycleGAN [72], GASDA involves both real to unreal and unreal to real image translations together with an epipolar geometry of the real stereo images. The network is trained with two image style translations and symmetric depth estimators to produce depth maps with 192×640 pixel resolution.

## 4. Evaluation Matrices and Criteria

The most commonly used quantitative metrics for evaluating the performance of monocular depth estimation methods are Absolute Relative Difference (AbsRel), Root Mean Square Error (RMSE), RMSE (log) and Square Relative Error (SqRel).

These metrics are defined as follows:

(2) $$\mathrm{AbsRel}=\frac{1}{N}\sum \frac{\left|d_{i}-d_{i}^{*}\right|}{d_{i}}$$

(3) $$\mathrm{RMSE}=\sqrt{\frac{1}{N}\sum {\left|d_{i}-d_{i}^{*}\right|}^{2}}$$

(4) $$\mathrm{RMSE}(\log)=\sqrt{\frac{1}{N}\sum {\left|\log d_{i}-\log d_{i}^{*}\right|}^{2}}$$

(5) $$\mathrm{SqRel}=\frac{1}{N}\sum \frac{{\left|d_{i}-d_{i}^{*}\right|}^{2}}{d_{i}}$$

(6) $$Accuracy\;with\;threshold\;\left(\delta <thr\right):\%\;of\;d_{i}\;such\;that\max\left(\frac{d_{i}}{d_{i}^{*}},\frac{d_{i}^{*}}{d_{i}}\right)<thr,\;where\;thr=1.25,1.25^{2},1.25^{3}$$

where $d_{i}$ and $d_{i}^{*}$ are the ground truth and predicted depth at pixel i and N is the total number of pixels.

All of the methods described in this section are tested on either KITTI [31] or NYU-v2 [29] datasets. In order to evaluate and compare all the methods, we used the publicly available pre-trained models. The main advantage of comparing the pre-trained models on both datasets is that it allows us to measure the generalised performance of the networks on different test sets. Table 3 illustrates the properties of the networks studied for monocular depth estimation including their input/output dimensions, number of parameters, Graphical Processing Unit (GPU) specification and the type of the architecture employed.

Table 4 presents the performance evaluation of the studied methods on KITTI [31] dataset. All the numbers presented in this table are reported by the respective authors. As shown in Table 4, DeepV2D [50] marginally achieved the best accuracy on the KITTI [31] dataset. The last four columns in this table represent the evaluation using RMSE (log) metric and threshold inlier measures defined in Equation (6). Not all the methods in Table 4 are trained and evaluated on the same part of the KITTI [31] dataset. The Train and Test columns in Table 4 indicate the subsets of the KITTI [31] dataset used by each method.

In another evaluation on the NYU-v2 [29] dataset, as shown in Table 5, DeepV2D [50] marginally achieved the best accuracy with very close performance to BTS [49]. The significant advantage of this method against the state-of-the-art is a learnable approach for a geometrical principal of structure from motion and relative camera pose estimation.

Note that, some of the methods in Table 5 such as monodepth2 [42] and PackNet-SfM [52] are only trained and evaluated on KITTI-ES(RD) as reported in their original papers. To achieve a fair and generalized comparison, we evaluated LSIM [51], PackNet-SfM [52], GASDA [54], VOMonodepth [53] and monodepth2 [42] on the NYU-v2 dataset [29]. The numbers for the rest of the methods are reported by the respective authors.

Table 6 compares the performances of the studied methods in terms of inference time. As shown in Table 6, BTS [49] has the fastest inference time with 0.22 s.

An additional set of methods are studied and compared as presented in Appendix A. These methods are evaluated on either KITTI [31] or NYU-v2 [29] datasets and the comparison includes the parameter counts, depth accuracy measured using RMSE metric, memory requirement and training environment. All the methods in Appendix A, Table A1 are compared with the state-of-the-art monocular depth estimation methods. These methods are categorized as of low accuracy with expensive computational time and slow convergence rate which led us to exclude them from this survey.

**Figure 1:**
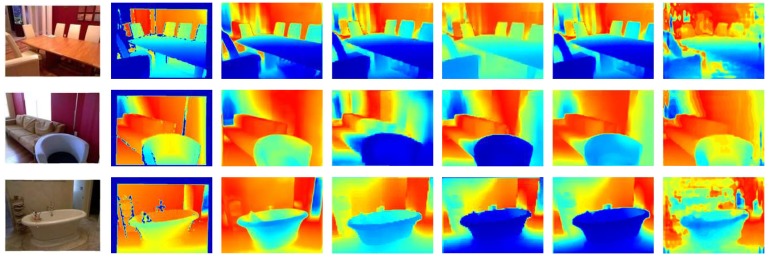
Qualitative comparison of five state-of-the-art-monocular depth estimation methods. From left to right: Input Image, Ground Truth, BTS [49], DeepV2D [50], DenseDepth [47], MonoResMatch [38] and DORN [18].

Due to the technical complications with the publicly available codes and lack of instructions, we were not able to test all 13 methods for qualitative comparisons. Only five methods were implemented successfully and validated on NYU-v2 [29] dataset. A few samples of the results are illustrated in Figure 1. This visual comparison also supports the claim from the previous tables that DeepV2D [50] marginally outperforms BTS [49] and other methods as it can estimate smoother depth maps with sharper boundaries, less artifacts and relative scale.

## 5. Discussion

Monocular depth estimation plays a crucial role in understanding 3D scene geometry in many applications. A single 2D image may be produced from an infinite number of distinct 3D scenes, which is a classical monocular depth estimation approach. The classical monocular depth estimation methods utilise meaningful monocular cues, such as perspective and texture information, objects size, object locations and occlusions, resulting in an undesirable low-resolution depth prediction. Recently, deep learning methods significantly improved the performance of the monocular depth estimation methods by exploring image-level information and hierarchical features in the network. However, these methods employ repeated spatial pooling operations. To obtain high-resolution depth maps, skip connection-based networks are required, however, these methods tend to make the training process complicated and require more computational time. To target these issues, CNN based transfer learning methods were employed resulting in high-quality depth estimation. In general, deep-learning methods achieved outstanding results, however, they require a large amount of data labelled with precise depth measurements for training. The introduction of different methodologies and architectures such as local planar guidance layers (LPGL), multi-layer deconvolutional networks and atrous spatial pyramid have moved the performance of these models to the next level.

### 5.1. Comparison Analysis Based on Performance

**I. Degree of supervision:** most of the methods demonstrated in this paper require ground truth depth images for training. These supervised methods perform well and most of them are state-of-the-art on common benchmarks. Methods such as DeepV2D [50], BTS [49] and VNL [48] showed a much faster performance time compared to the other models. On the other hand, VNL [48], ACAN [46] and EMDEOM [32] provides the depth information with much lower resolution compared to the state-of-the-art. Unlike VNL [48], DORN [18] has the highest number of parameters in the supervised category and it requires a high number of operations making it an inefficient choice for real-life applications.

Obtaining large datasets of RGB images with accurate ground truth depth images is a challenging task. As such, methods that do not require full supervision (labelled ground truth) are more attractive. Methods such as LISM [51], monoResMatch [38], PackNet-SfM [52] and monodepth2 [42] are self-supervised methods. Although most of these methods can generate high resolution depth maps with comparable accuracy against the state-of-the-art, they are computationally expensive and require a significant amount of memory.

**II. Accuracy and depth range:** based on our evaluations, DeepV2D [50] marginally achieved the best performance compared to BTS [49] and the rest of the methods. On KITTI [31] dataset the model achieved 2.005 RMSE and threshold accuracy of 0.977 with $\delta <1.25^{3}$. On NYUD-v2 [29] dataset it achieved 0.403 RMSE and threshold accuracy of 0.996 with $\delta <1.25^{3}$. As shown in Table 4 and Table 5, methods with 3D geometry constraint or features, outperform the others, which shows the importance of high order 3D geometric constraints for depth estimation.

The evaluation of BTS [49], DORN [18], VNL [48], DenseDepth [47] and VOMonodepth [53] indicated that supervised learning approaches achieved better results compared to semi and self-supervised methods.

**III. Computation time and memory:** based on the comparisons presented in Table 3, Table 4, Table 5 and Table 6, VNL [48] significantly reduced the computational time and memory footprint, which can be used for both quality and low-cost monocular depth estimation.

The advancement of deep-learning methodologies suggests that cameras may become a competitive source of reliable 3D information. Compared to the conventional method, these models have the potential to be optimised for deployment on smart and consumer platforms.

These methods are composed in two ways: feature extraction which is done in encoder part using the powerful pre-trained models such as VGG [67], ResNet [57] or DenseNet [61], while the desired depth prediction is obtained using the decoder network architecture.

### 5.2. Future Research Directions

Over the past couple of years, deep-learning approaches have shown a significant improvement in the performance of monocular depth estimation. The topic is still in its infancy and further developments are yet to be expected. In this section, we present some of the current directions and issues for further future research.

1. Complex deep networks are very expansive in terms of memory requirements, which is a major issue when dealing with high-resolution images and when aiming to predict high-resolution depth images.
2. Developments in high-performance computing can address the memory and computational issues, however, devolving lighter deep network architectures remains desirable especially if it is to be deployed in smart consumer devices.
3. Another challenge is how to achieve higher accuracy, in general, which is affected by the complex scenarios, such as occlusions, highly cluttered scenes and complex material properties of the objects.
4. Deep-learning methods rely heavily on the training datasets annotated with ground truth labels for depth estimation which is very expansive to obtain in the real world.
5. We expect in the future to see the emergence of large databases for 3D reconstruction. Emerging new self-adoption methods that can adapt themselves to new circumstances in real-time or with minimum supervision are one of the promising future directions for research in depth estimation.

This paper provided a preliminary review of the recent developments in monocular depth estimation using deep-learning models. Regardless of its infancy, these methods are achieving promising results, and some of these methods are competing, in terms of accuracy of the results, with the traditional methods. We have entered a new era where deep learning and data-driven techniques play an important role in image-based depth estimation.

## Figures and Tables

**Table 1:** Datasets for monocular depth estimation.

Dataset	Labelled Images	Annotation	Brief Description
NYU-v2 [29]	1449	Depth + Segmentation	Red-green-blue (RGB) and depth images taken from indoor scenes.
Make3D [30]	534	Depth	RGB and depth images taken from outdoor scenes.
KITTI [31]	94K	Depth aligned with RAW data + Optical Flow	RGB and depth from 394 road scenes.
Pandora [35]	250K	Depth + Annotation	RGB and depth images.
SceneFlow [36]	39K	Depth + Disparity + Optical Flow+ Segmentation Map	Stereo image sets rendered from synthetic data with ground truth depth, disparity and optical flow.

**Table 2:** Categories of deep learning-based monocular depth estimation methods (FC: fully convolutional; CNN: convolutional neural networks).

Method	Architecture	Category
EMDEOM [32]	FC	Supervised
ACAN [46]	Encoder-Decoder	
DenseDepth [47]	Encoder-Decoder	
DORN [18]	CNN	
VNL [48]	Encoder-Decoder	
BTS [49] DeepV2D [50]	Encoder-Decoder CNN	
LISM [51]	Encoder-Decoder	Self-supervised
monoResMatch [38]	CNN	
PackNet-SfM [52]	CNN	
VOMonodepth [53]	Auto-Decoder	
monodepth2 [42]	CNN	
GASDA [54]	CNN	Semi-supervised

**Table 3:** Properties of the studied methods for monocular depth estimation (FC: fully convolutional; ED: encoder-decoder; AD: auto-decoder; CNN: convolutional neural networks; K: trained on KITTI; N: trained on NYU-v2).

Method	Input	Type	Optimizer	Parameters	Output	GPU Memory	GPU Model
BTS [49]	352× 704 K	ED	Adam	47M	352× 704 K	4×11 GB	1080 Ti
DORN [18]	385×513 K	CNN	Adam	123.4M	513 × 385 K	12 GB	TITAN Xp
VNL [48]	384× 384 N	ED	SGD	2.7M	384 × 384 N	N/A	N/A
ACAN [46]	256× 352 N	ED	SGD	80M	256 × 352 N	11 GB	1080 Ti
VOMonodepth [53]	256 × 512 K	AD	Adam	35M	256×512 K	12 GB	TITAN Xp
LSIM [51]	1242 × 375 K	ED	Adam	73.3M	1242 × 375 K	12 GB	TITAN Xp
GASDA [54]	192 × 640 K	CNN	Adam	70M	192 × 640 K	N/A	N/A
DenseDepth [47]	640 × 480 N	ED	Adam	42.6M	320 × 240 N	4×12 GB	TITAN Xp
monoResMatch [38]	192 × 640 K	CNN	Adam	42.5M	192 × 640 K	12 GB	TITAN Xp
EMDEOM [32]	304 × 228 K	FC	Adam	63M	128 × 160 K	12 GB	TITAN Xp
PackNet-SfM [52]	640 × 192 K	CNN	Adam	128M	640 × 192 K	8×16 GB	Tesla V100
monodepth2 [42] DeepV2D [50]	640× 192 K 640 × 480 N	CNN CNN	Adam RMSProp	70M 32M	640 × 192 K 640 × 480 N	12 GB 11 GB	TITAN Xp 1080 Ti

**Table 4:** Evaluation results on KITTI dataset. Best method per metric is emboldened and highlighted in green. (RD: KITTI Raw Depth [31]; CD: KITTI Continuous Depth [31,32]; SD: KITTI Semi-Dense Depth [31,32]; ES: Eigen Split [33]; ID: KITTI Improved Depth [34]).

Method	Train	Test	Abs Rel	Sq Rel	RMSE	RMSElog	δ<1.25	$\delta <1.25^{2}$	$\delta <1.25^{3}$
BTS [49]	ES(RD)	ES(RD)	0.060	0.182	**2.005**	0.092	0.959	**0.994**	**0.999**
DORN [18]	ES(RD)	ES(RD)	0.071	0.268	2.271	0.116	0.936	0.985	0.995
VNL [48]	ES(RD)	ES(RD)	0.072	0.883	3.258	0.117	0.938	0.990	0.998
ACAN [46]	ES(RD)	ES(RD)	0.083	0.437	3.599	0.127	0.919	0.982	0.995
VOMonodepth [53]	ES(RD)	ES(RD)	0.091	0.548	3.790	0.181	0.892	0.956	0.979
LSIM [51]	FT	RD	0.169	0.6531	3.790	0.195	0.867	0.954	0.979
GASDA [54]	ES(RD)	ES(RD)	0.143	0.756	3.846	0.217	0.836	0.946	0.976
DenseDepth [47]	ES(RD)	ES(RD)	0.093	0.589	4.170	0.171	0.886	0.965	0.986
monoResMatch [38]	ES(RD)	ES(RD)	0.096	0.673	4.351	0.184	0.890	0.961	0.981
EMDEOM [32]	RD, CD	SD	0.118	0.630	4.520	0.209	0.898	0.966	0.985
monodepth2 [42]	ES(RD)	ES(RD)	0.115	0.903	4.863	0.193	0.877	0.959	0.981
PackNet-SfM [52]	ES(RD)	ID	0.078	0.420	3.485	0.121	0.931	0.986	0.996
DeepV2D [50]	ES(RD)	ES(RD)	**0.037**	**0.174**	**2.005**	**0.074**	**0.977**	0.993	0.997

**Table 5:** Evaluation results on NYU-v2 dataset. Best method per metric is emboldened and highlighted in green.

Method	Abs Rel	Sq Rel	RMSE	RMSElog	δ<1.25	$\delta <1.25^{2}$	$\delta <1.25^{3}$
BTS [49]	0.112	**0.025**	**0.352**	0.047	0.882	0.979	0.995
VNL [48]	0.113	0.034	0.364	0.054	0.815	**0.990**	0.993
DenseDepth [47]	0.123	0.045	0.465	0.053	0.846	0.970	0.994
ACAN [46]	0.123	0.101	0.496	0.174	0.826	0.974	0.990
DORN [18]	0.138	0.051	0.509	0.653	0.825	0.964	0.992
monoResMatch [38]	1.356	1.156	0.694	1.125	0.825	0.965	0.967
monodepth2 [42]	2.344	1.365	0.734	1.134	0.826	0.958	0.979
EMDEOM [32]	2.035	1.630	0.620	1.209	0.896	0.957	0.984
LSIM [51]	2.344	1.156	0.835	1.175	0.815	0.943	0.975
PackNet-SfM [52]	2.343	1.158	0.887	1.234	0.821	0.945	0.968
GASDA [54]	1.356	1.156	0.963	1.223	0.765	0.897	0.968
VOMonodepth [53]	2.456	1.192	0.985	1.234	0.756	0.884	0.965
DeepV2D [50]	**0.061**	0.094	0.403	**0.026**	**0.956**	0.989	**0.996**

**Table 6:** Comparison of the models in terms of inference time (FC: fully convolutional; CNN: convolutional neural networks). Best method is emboldened and highlighted in green.

Method	Inference Time	Network/FC/CNN
BTS [49]	0.22 s	Encoder-decoder
VNL [48]	0.25 s	Auto-decoder
DeepV2D [50]	0.36 s	CNN
ACAN [46]	0.89 s	Encoder-decoder
VOMonodepth [53]	0.34 s	CNN
LSIM [51]	0.54 s	CNN
GASDA [54]	0.57 s	Encoder-decoder
DenseDepth [47]	0.35 s	Encoder-decoder
monoResMatch [38]	0.37 s	CNN
EMDEOM [32]	0.63 s	FC
DORN [18]	0.98 s	Encoder-decoder
PackNet-SfM [52]	0.97 s	CNN
monodepth2 [42]	0.56 s	CNN

## Appendix A

### Low-Performance Monocular Depth Estimation Methods

Table A1 summarizes the monocular depth estimation methods in terms of parameter counts, depth accuracy measured using RMSE metric, memory requirement and training environment. These methods are categorized as low accuracy with slow convergence rate and are excluded from this survey. All the numbers presented in this table are reported by the respective authors.

**Table A1:** Properties of the low-accuracy methods trained on either KITTI or NYU-v2 datasets. (FC: fully convolutional, ED: encoder-decoder, AD: auto-decoder, K: trained on KITTI dataset, N: trained on NYU-v2 dataset and CNN: convolutional neural networks).

Method	Input	Type	Optimizer	Parameters	Output	GPU Memory	RMSE	GPU Model
Zhou et al. [70]	128× 416 K	CNN	Adam	N/A	128× 416 K	N/A	4.975	N/A
Casser et al. [73]	128 × 416 K	CNN	Adam	N/A	128× 416 K	11 GB	4.7503	1080 Ti
Guizilini et al. [74]	640× 192 K	FC	Adam	86M	640 × 192 K	N/A	4.601	N/A
Godard et al. [15]	640× 192 K	FC	Adam	31M	640 × 192 K	12 GB	4.935	TITAN Xp
Eigen et al. [33]	640× 184 K	CNN	Adam	N/A	640 × 184	6 GB	N/A	TITAN Black
Guizilin et al. [75]	640× 192 K	ED	Adam	79M	640 × 192	8 × 16 GB	4.270	Tesla V100
Tang et al. [76]	640× 192 K	CNN	RMSprop	80M	640× 192	12 GB	N/A	N/A
Ramamonjisoa et al. [40]	640× 480 N	ED	Adam	69M	640× 480 N	11 GB	0.401	1080 Ti
Riegler et al. [39]	N/A	ED	Adam	N/A	N/A	N/A	N/A	N/A
Ji et al. [37]	320× 240 N	ED	Adam	N/A	320× 240 N	12 GB	0.704	TITAN Xp
Almalioglu et al. [77]	128× 416 K	GAN	RMSprop	63M	128× 416 K	12 GB	5.448	TITAN V
Pillai et al. [41]	128× 416 K	CNN	Adam	97M	128× 416 K	8 × 16 GB	4.958	Tesla V100
Wofk et al. [24]	224× 224 N	ED	SGD	N/A	224× 224 N	N/A	0.604	N/A
Watson et al. [78]	128× 416 K	ED	SGD	N/A	128× 416 K	N/A	N/A	N/A
Chen et al. [79]	256 × 512 K	ED	Adam	N/A	256× 512 K	11 GB	3.871	1080 Ti
Lee et al. [80]	640× 480 N	CNN	SGD	61M	640× 480 N	N/A	0.538	N/A
